# Pilot study on high-resolution radiological methods for the analysis of cerebrospinal fluid (CSF) shunt valves

**DOI:** 10.1016/j.zemedi.2023.11.001

**Published:** 2023-12-15

**Authors:** Martin P. Pichotka, Moritz Weigt, Mukesch J. Shah, Maximilian F. Russe, Thomas Stein, T. Billoud, Jürgen Beck, Jakob Straehle, Christopher L. Schlett, Dominik v. Elverfeldt, Marco Reisert

**Affiliations:** a Division of Medical Physics, Department of Diagnostic and Interventional Radiology, University Medical Center Freiburg, Faculty of Medicine, University of Freiburg, Freiburg, Germany; b Department of Neurosurgery, Medical Center – University of Freiburg, Faculty of Medicine, University of Freiburg, Freiburg, Germany; c Department of Diagnostic and Interventional Radiology, Medical Center – University of Freiburg, Faculty of Medicine, University of Freiburg, Freiburg, Germany; d Department of Stereotactic and Functional Neurosurgery, Department of Diagnostic and Interventional Radiology, Medical Physics, Medical Center of the University of Freiburg, Medical Faculty of the University of Freiburg, Germany

**Keywords:** Cerebrospinal fluid shunt valves, CSF, Shuntography, Photon-counting CT, Digital subtraction radiography, Machine learning

## Abstract

**Objectives:**

Despite their life-saving capabilities, cerebrospinal fluid (CSF) shunts exhibit high failure rates, with a large fraction of failures attributed to the regulating valve. Due to a lack of methods for the detailed analysis of valve malfunctions, failure mechanisms are not well understood, and valves often have to be surgically explanted on the mere suspicion of malfunction.

The presented pilot study aims to demonstrate radiological methods for comprehensive analysis of CSF shunt valves, considering both the potential for failure analysis in design optimization, and for future clinical in-vivo application to reduce the number of required shunt revision surgeries. The proposed method could also be utilized to develop and support in situ repair methods (e.g. by lysis or ultrasound) of malfunctioning CSF shunt valves.

**Materials and methods:**

The primary methods described are contrast-enhanced radiographic time series of CSF shunt valves, taken in a favorable projection geometry at low radiation dose, and the machine-learning-based diagnosis of CSF shunt valve obstructions. Complimentarily, we investigate CT-based methods capable of providing accurate ground truth for the training of such diagnostic tools. Using simulated test and training data, the performance of the machine-learning diagnostics in identifying and localizing obstructions within a shunt valve is evaluated regarding per-pixel sensitivity and specificity, the Dice similarity coefficient, and the false positive rate in the case of obstruction free test samples.

**Results:**

Contrast enhanced subtraction radiography allows high-resolution, time-resolved, low-dose analysis of fluid transport in CSF shunt valves. Complementarily, photon-counting micro-CT allows to investigate valve obstruction mechanisms in detail, and to generate valid ground truth for machine learning-based diagnostics.

Machine-learning-based detection of valve obstructions in simulated radiographies shows promising results, with a per-pixel sensitivity >70%, per-pixel specificity >90%, a median Dice coefficient >0.8 and <10% false positives at a detection threshold of 0.5.

**Conclusions:**

This ex-vivo study demonstrates obstruction detection in cerebro-spinal fluid shunt valves, combining radiological methods with machine learning under conditions compatible to future in-vivo application.

Results indicate that high-resolution contrast-enhanced subtraction radiography, possibly including time-series data, combined with machine-learning image analysis, has the potential to strongly improve the diagnostics of CSF shunt valve failures. The presented method is in principle suitable for in-vivo application, considering both measurement geometry and radiological dose. Further research is needed to validate these results on real-world data and to refine the employed methods.

In combination, the presented methods enable comprehensive analysis of valve failure mechanisms, paving the way for improved product development and clinical diagnostics of CSF shunt valves.

## Introduction

In the following we present a pilot study on novel radiological methods for comprehensive functional diagnostics of Cerebro-Spinal Fluid (CSF) shunt valves (CSF-SVs). The presented methods are in principle applicable to clinical diagnostics of CSF shunt valve patency, researching recovery methods for occluded valves, and designing shunt valves more resilient to failure.

CSF shunts are the standard treatment for hydrocephalus, a condition affecting regulation of CSF pressure in the brain, with a prevalence of ∼0.5% of the overall population [1]. High failure rates (40% within 2 years, 98% within 10 years) of these CSF shunt devices lead to high mortality rates and massive costs to the healthcare system due to shunt revisions and related procedures [2], [3]. In posthaemorrhagic hydrocephalus, reported CSF shunt revision rates are as high as 52%, with 45% of patients requiring surgical revision within the first 6 months of implantation. Obstruction by coagulated blood is a common cause of shunt failure [4], [5], [6], [7]. Many shunt obstructions (∼30% [2]) occur within the valve itself. Despite ongoing research, no major producer provides solutions to measure CSF flow noninvasively [3].

A recent approach in clinical CSF shunt radiography are “contrast enhanced shunt series”, outperforming head CT and methods based on radionuclide markers in terms of predictive value and spatiotemporal resolution [8]. However, with this approach the level of detail observed within the valve itself is low, preventing detailed analysis of its condition.

**Figure 1:**
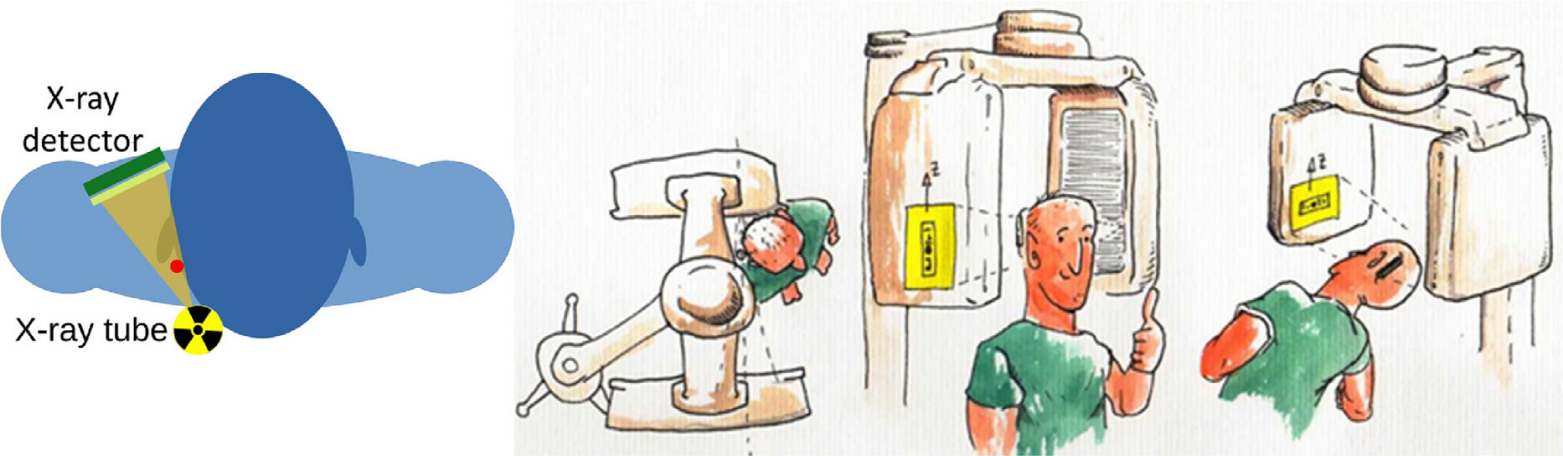
Left: Schematics of a clinically viable high magnification radiographic acquisition geometry. Possible patient positionings in a dental Cone Beam CT scanner are illustrated on the right. To check the patency of the gravitational lock, measurements under inclincation can be performed.

Fortunately, the exposed location of CSF-SVs within the patient enables a favorable projection geometry, where radiography can be performed tangentially to the skull surface at relevant geometrical magnification. This is depicted in Fig. 1.

With accurate collimation, the expected radiological dose of this procedure is anticipated to be low, as only a small portion of soft tissue needs to be penetrated in this geometry.

This possibility raises the question whether reliable diagnostics of CSF-SVs can be performed with high resolution radiographs from a single view angle. The current study addresses possibilities to generate ground truth data for failure states and to detect valve occlusions from digital subtraction radiographies, forming the basis for such diagnostics.

## Materials and methods

### Contrast-enhanced radiographic flow measurements of CSF-SVs

**Figure 2:**
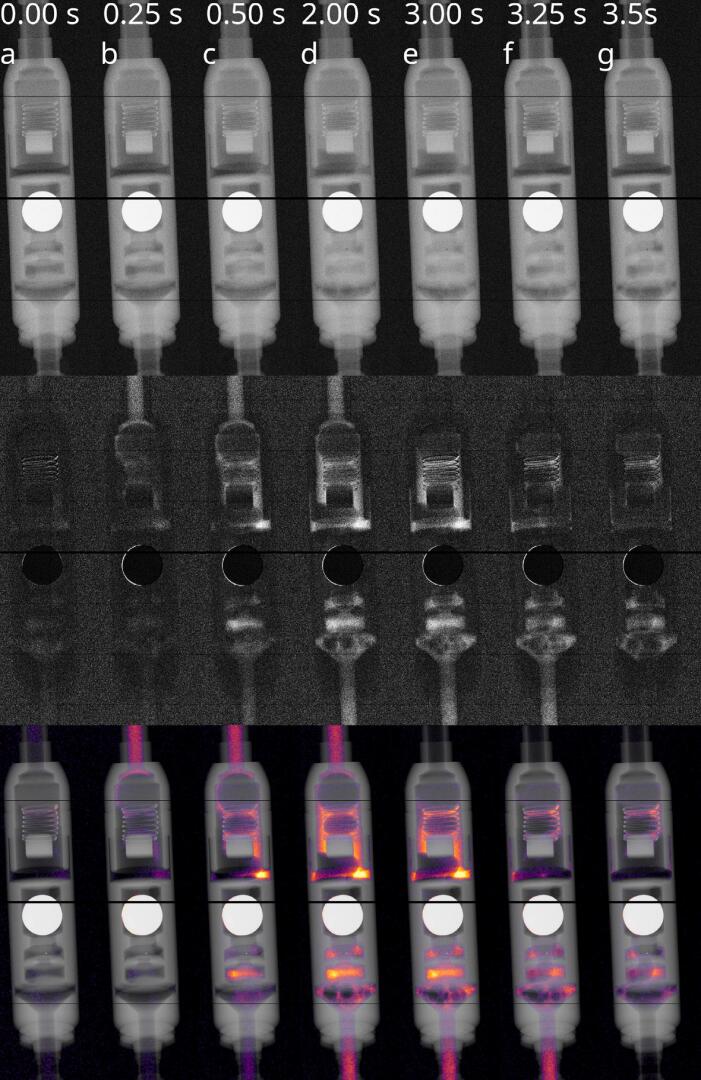
Selected frames of a radiographic time series taken at 20 Hz after injection of a .4 ml bolus of Gadovist 1 mmol/ml. Detailed measurement parameters can be found in the Supplemental Digital Content. The background attenuation is depicted in grey, whereas the contrast agent amplitude is color-coded using the “Inferno” colormap. a) Valve filled with water, b,c) contrast bolus enters valve from top d) Valve completely filled with contrast agent, air bubbles visible in lower part e,f) Water pushes out contrast agent from top g) Valve flushed, some contrast agent remains. Top: Normalized radiographies; black: 100% transmission, white: 0% transmission. Middle: Difference to radiography without contrast agent. Black: Absorption difference 0%, white: absorption difference 20%. Bottom: Difference 5 × 5 × 5 median-filtered and color-coded, overlaid with radiography.

By injection of contrast agent boli, fluid transport within the valve can be assessed in radiographic series with a high level of detail in the proposed acquisition geometry. For the measurements shown in Fig. 2 the source to object and detector distances were chosen at 5.5 cm and 35 cm respectively, representing a clinically viable geometry. The corresponding measurement parameters are: X-ray source: Hamamatsu L8601-01, operated at 90 kVp with a target current of 110 μA, .5 mm Al, detector: DECTRIS EIGER2X CdTe 1M-W, 2048 × 512 pixels, pixel pitch 75 μm, energy thresholds set at 45 kV & 55 kV. The shunt system was depleted with physiological NaCl solution prior the measurement. At a base pressure of 20 mbar, a .4 ml bolus of Gd-based contrast agent (Gadovist 1 mmol/ml; 157 mg/ml) was injected. Compared to e.g. routine MRI perfusion measurements, this is a very small contrast agent dose [9]. The detector utilized features 8 × 2 individual detector tiles, with major gaps (10 pixels in the center, 3 pixels for the first and last quarter) visible as horizontal stripes in Fig. 2. The air bubbles visible here are avoidable by improved experimental designs. The dose load for the entire radiographic series, determined by thermo-luminescent MCP-N dosimeters, was ∼5 µSv. A corresponding video can be found in the Supplemental Digital Content, Video 1.

A different entity that can be determined directly from such high-resolution radiographs is the spring compression at the valve inlet (see Fig. 5). Correlating the pressure at the valve entrance to the spring compression provides complementary means to assess the valve condition. Similarly, patency of the gravitational unit, which adapts the opening pressure with respect to the patient orientation, can be determined by tracking movement of the gravitational unit with inclination.

### Tomographic analysis of CSF shunt valves

Radiographically obtained flow patterns can be compared to a model of the shunt valve’s cavity, where contrast agent flow is expected in absence of obstructions. This information can be provided by tomographic analysis of the valves, followed by 3D–2D alignment of the generated morphological models to the pose of the valve observed in radiography. Alignment of the radiographic data with the model data is best achieved by landmark-based co-registration, since CSF-SVs exhibit prominent features suitable for landmark-based image alignment.

However, due to high attenuation contrast (in case of the valves shown here: metallic outer shell, spring, ruby and tantalum balls), CSF-SVs are challenging in CT imaging. Beam-hardening causes strong image artifacts, hindering the generation of accurate 3D models if not addressed properly. For this reason, we employed photon-counting CT (PCCT), which recently was introduced to the clinical routine and allows to efficiently suppress beam-hardening artifacts.

### Automated diagnostics of shunt valve obstructions

We utilized synthetic projections to evaluate the possibility of automated valve diagnostics via machine learning (U-Net). Based on a segmented PCCT dataset of a CSF-SV, the cavity volume of the valve was virtually filled with Gd-based contrast agent, with the exception of randomly placed “organic depositions”, which were assumed to be at water density. The model was projected forward using realistic binomial photon statistics. The projected mask of the obstruction was provided to the network in combination with the artificial projections of the valve as training data.

We employed the patchwork architecture [10], hierarchical, multiscale U-Net based framework to segment the defects. The approach involves using nested patches of a fixed matrix size that decrease in physical size. A U-Net-type architecture was utilized in each scale, with the matrix size set at 128 × 128 pixels for all scales. A scale pyramid with a depth of four was employed. The architecture of the U-Net used follows [11], with feature dimensions (32,32,64,64,128) and max-pooling and transposed convolutions in the encoding and decoding layers, respectively. The network was trained with the Adam optimizer with a rate of 0.001 and batch size 32. No systematic tuning was performed, and all labels were trained using binary-crossentropy.

**Figure 3:**
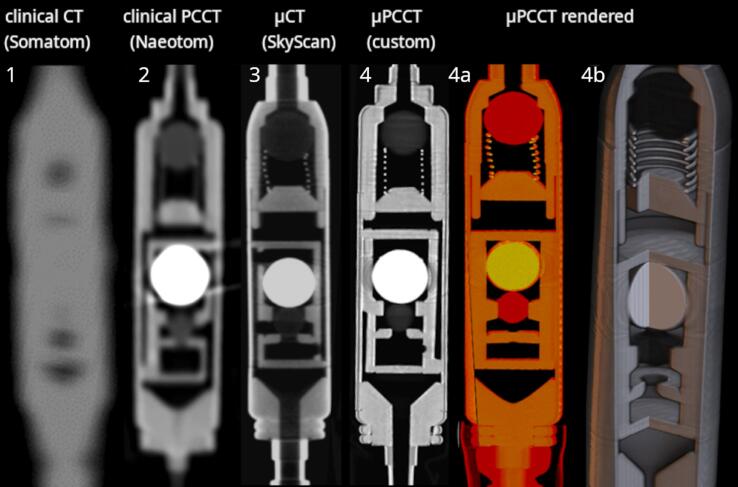
Cross-sections of Miethke GAV CSF shunt valves obtained from different CT scanners. From left to right: Siemens Somatom Force (EID, kernel: Br64, algorithm: Admire level 3), Siemens Naeotom (Ultra High-Resolution mode, kernel: Hr80, algorithm: QIR level 3), Bruker SkyScan 1276 (100 kVp, Cu .1 mm) and our custom µPCCT system (90 kVp, Al 2 mm). The renderings on the right (4a&b) illustrate a high level of detail and accurate reproduction of lower attenuation structures, e.g. the ruby balls, visible in red in 4a.

Assuming well controlled experimental parameters, the U-Net was trained with fixed values for open beam counts (400cts) and contrast agent concentration (2 mmol/ml), assuming randomly distributed sizes of the obstructions up to 0.9 mm^3^. The total number of training images was 2500, the number of test images 5000, namely 2500 without obstruction and 2500 with obstruction. These projections were generated by adding virtual obstructions of random placement and volume (0.025–0.9 mm^3^ in volume) to the base volume of a single reconstructed CSF shunt valve (see Fig. 3, item 4). Each projection was cast, including binomial noise statistics, at a random rotational angle about the long axis of the valve, which is the relevant degree of freedom in in-vivo application. Overall training time of the U-Net on an NVIDIA RTX6000 was about 4 h (stopped after saturation of training error).

## Results

### Tomographic analysis

Fig. 3 compares the imaging performance of state-of-the-art energy integrating CT (cross sections 1 & 3 from the left) in case of CSF-SVs to PCCT (cross sections 2 & 4). The µCT data were reconstructed by a custom OSEM type iterative reconstruction with 5 iterations and 16 subsets. Signal-to-thickness (STC) calibration was used to pre-process the µPCCT projections prior to reconstruction (here using Cu filters) [12], [13].

**Figure 4:**
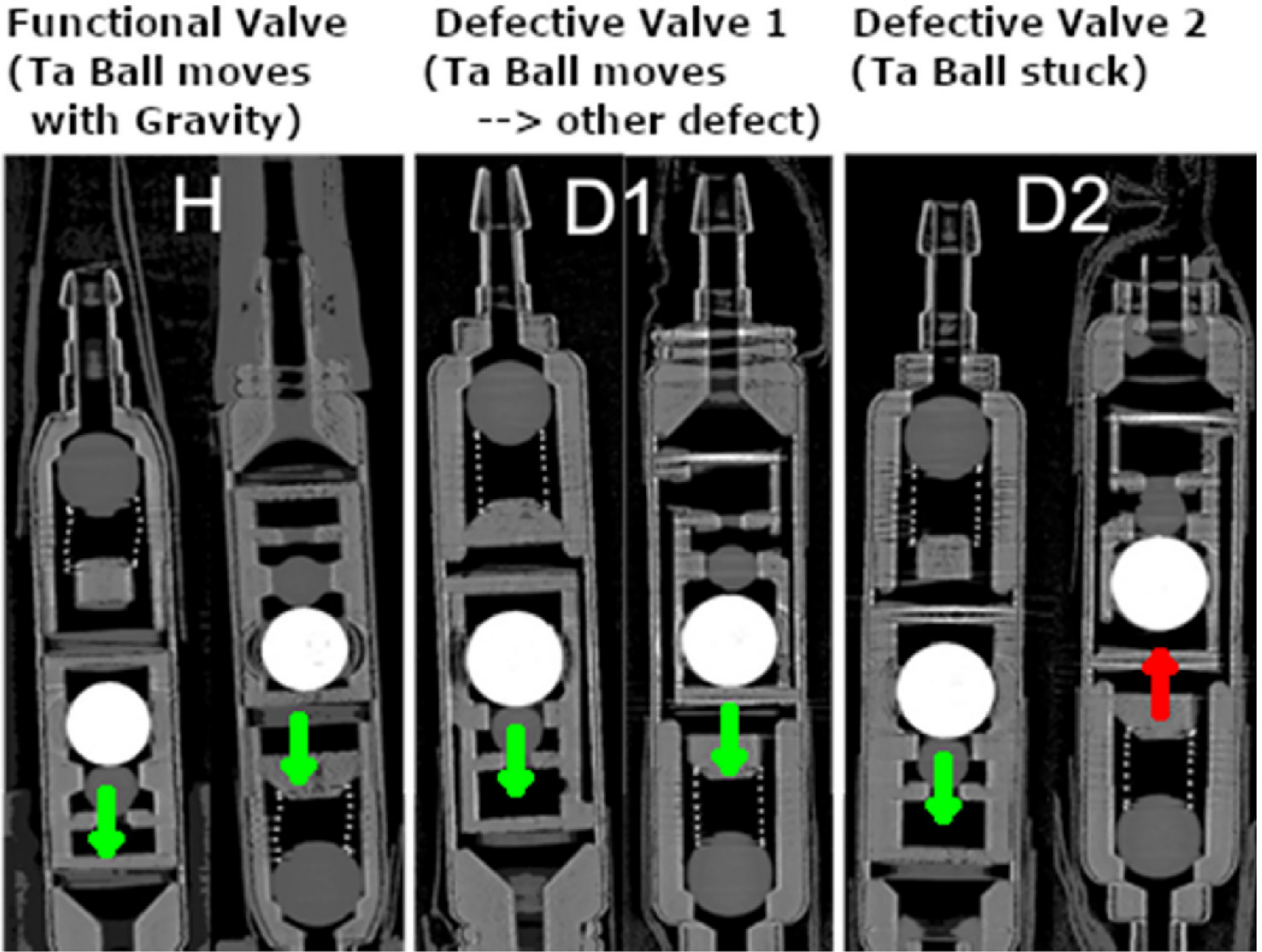
Investigation of the functionality of the gravity unit. 3 different shunt valves (H = healthy, D1/2 = defective 1/2) have been imaged in flipped positions, i.e. upright and rotated by 180 degrees. In case of D2 the gravitational unit appears defective.

µPCCT analysis was also employed to investigate valves which had been identified as non-functional in a pressure-flow setup of the department of neurosurgery of UMCF. Fig. 4 shows µPCCT cross-sections of one healthy (H) and two malfunctioning (D1/2) CSF-SVs, identified in this setup. To trigger the gravitational unit the orientation of the valves was inverted between the respective scans shown left and right. It can be observed that in case of D2 the gravitational unit is malfunctioning.

### Radiographic analysis

**Figure 5:**
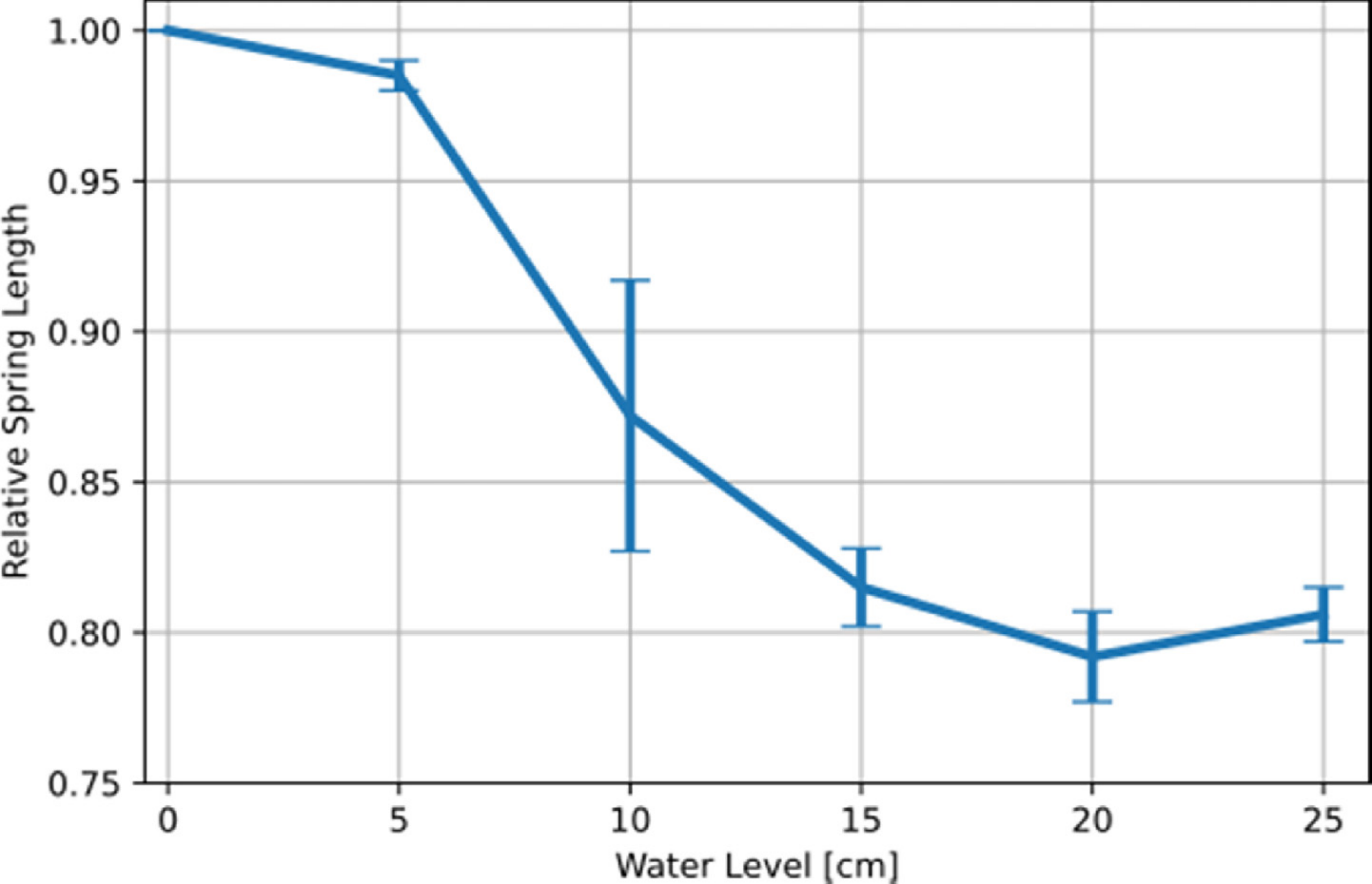
Tracking of the spring compression of a functional Miethke GAV 5/40 shunt under varying fluid pressure. For each position 12 data points were acquired. The error bars indicate the standard deviation. The apparent relaxation at 25 cm water level appears counter intuitive, but is within the standard deviation of the measurement.

In a next step we verified that the spring compression at the valve inlet could be tracked in the radiographic regime with the geometrical parameters discussed above. To this end we determined the longitudinal distance of 5 consecutive slopes of the spring under varying pressure at the valve inlet. For this a pressure-flow setup was used in overflow condition. A 50 ml syringe was used as upper fluid reservoir and the vertical distance was measured from the knee of the water surface to the upper bound of the valve inlet. A Y-connector was added to the tubing to inject a small contrast agent bolus. The tubing length below the Y-connector was extended to allow for the injection of the bolus to complete approx. 3 s prior to the bolus reaching the valve inlet. For visual verification we added a small amount of E133 blue coloring agent to the contrast agent bolus. Fig. 5 displays the corresponding observed spring compression of a Miethke GAV 5/40, oriented horizontally during measurement.

### Automated diagnostics of shunt valve obstructions

The following analysis aims to evaluate the possibility of automated machine-learning-based obstruction detection based on the ground truth provided by high-resolution contrast-agent subtraction tomography of the intact and occluded valves. As no occluded valves were available for our study, this was done on simulated data (see “Materials and Methods”).

In a preliminary step the dependency of the U-Net performance on the key experimental parameters, i.e. the contrast agent concentration and photon statistics, was analyzed. To this end the U-Net described above was trained and tested with mixed parameter sets, comprising 5 different sizes of obstructions × 5 different contrast agent concentrations × 5 different sets of photon statistics. These parameter values were equally distributed across a training and test dataset of 1250 projections, with 10 projections for each individual parameter set.

**Figure 6:**
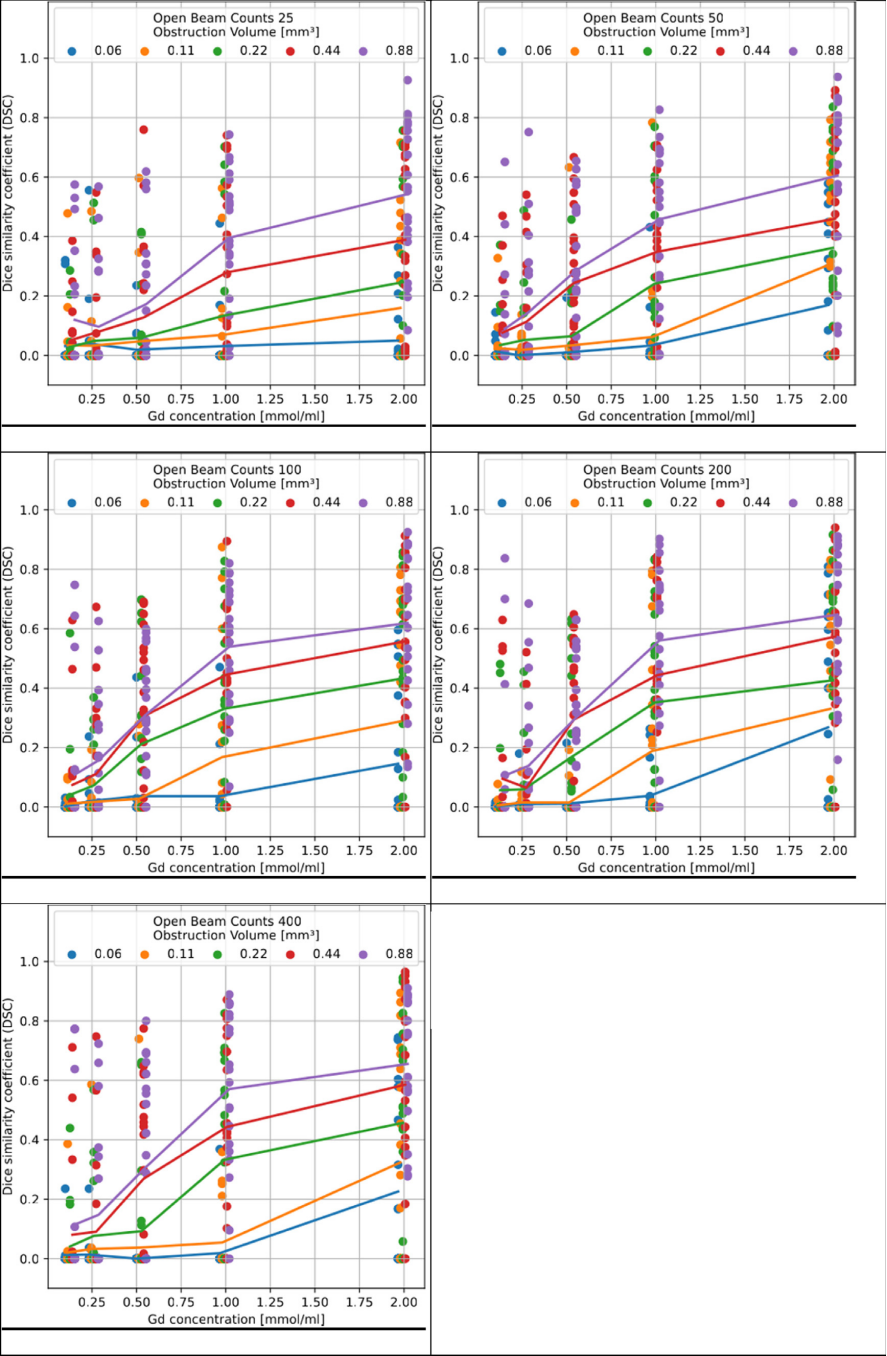
Response of U-Net detection under variation of contrast agent concentration, photon statistics and obstruction size.

Fig. 6 displays the response of U-Net detection (given as Dice similarity coefficient or DSC) under variation of contrast agent concentration, photon statistics (given as open beam counts) and obstruction size. A clear dependency on contrast agent concentration and obstruction size is observed.

**Figure 7:**
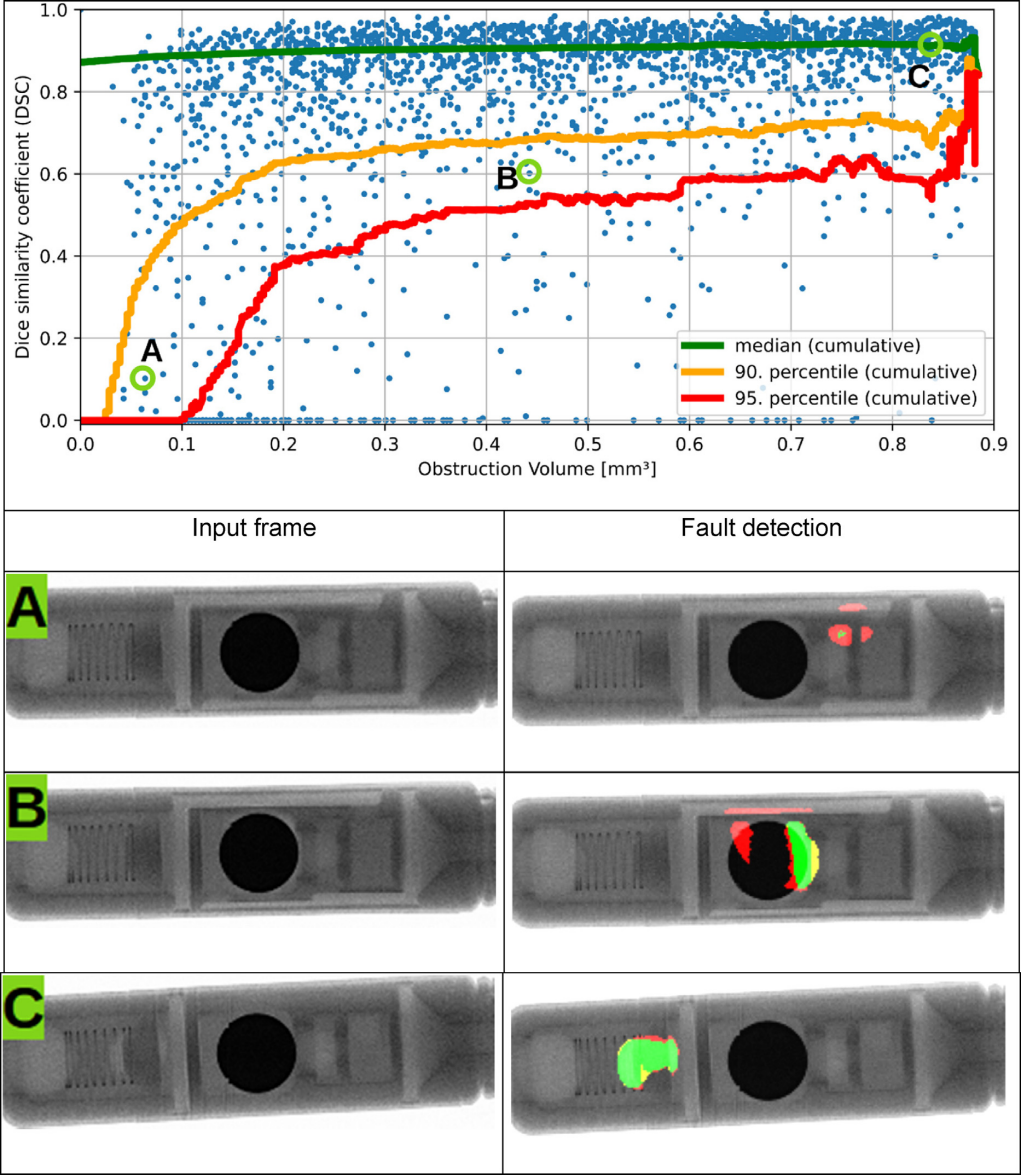
Top: Dice similarity coefficient for fixed open beam counts and contrast agent concentration. 2500 frames were used for training and 2500 frames were used for verification. Bottom: Exemplary detection of obstructions (as indicated above). Green indicates accurate detection; red indicates false negative and yellow indicates false positive. The grayscale of the input frame is 0–400 counts.

In a second step we generated a set of 7500 simulated projections (2500 for training and 5000 for testing), assuming a contrast agent concentration of 2 mmol/ml, 400 open beam counts and randomly distributed obstruction sizes up to 0.9 mm^3^ (as described in the materials and methods section). The achieved U-Net response under these conditions is shown in Fig. 7.

**Figure 8:**
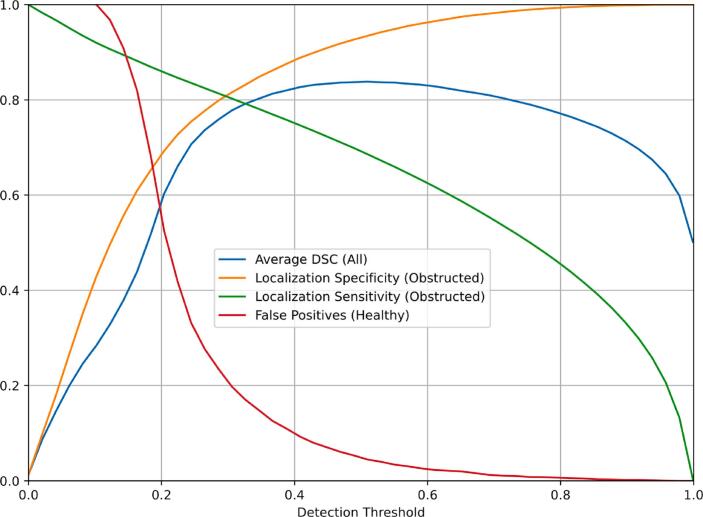
Dependency of DSC, specificity (correctly detected / overall detected pixels), sensitivity (correctly detected pixels / projected obstruction pixels) and false positive rate (threshold is 1 falsely detected pixel) on the detection threshold. Here 2500 frames with and 2500 without obstructions were tested. The Y-axis represents the measures given in the color-coded legend of the graph, i.e. the average DSC (blue), localization specificity (yellow), localization sensitivity (green), and false positive rate (red).

Shown in Fig. 8, the following measures were evaluated as a function of the detection threshold applied to the ouput of the U-Net: The localization specificity, sensitivity and Dice similarity index (DSC) were determined using 2500 simulated projections with randomly placed obstructions (see “Materials and Methods” for details), while the false positive rate was determined using 2500 virtual projections of the unobstructed valve. The Measures are defined as follows:

Sensitivity: (number of pixels correctly detected as obstruction / number of true obstruction pixels) averaged over the 2500 frames with obstructions.
Specificity: (number of pixels correctly detected as obstruction / number of pixels detected as obstruction) averaged over the 2500 frames with obstructions.
Dice similarity index (DSC): 2*(Sensitivity*Specificity) / (Sensitivity + Specificity) averaged over the 2500 frames with obstructions.
False Positive Rate: (number of unobstructed frames with detected obstruction / number of unobstructed frames) for the 2500 simulated frames without obstruction.

## Discussion

Fig. 3 clearly shows that, compared to conventional CT, PCCT provides a higher level of detail and contrast, as well as more homogeneous reproduction of constituent materials. Simultaneously, µCT (cross sections 3&4) provides significantly higher resolution compared to clinical devices (cross sections 1&2). Consequently, among the methods investigated µPCCT is best suited for ex-vivo analysis of CSF-SVs.

Figure 4, Figure 5 demonstrate that the mobility of the Tantalum ball functioning as a gravitational lock and the pressure-dependent compression of the valve inlet spring can be clearly resolved by our imaging methods. Analysis of spring compression and ball mobility under different tilting angles could further support the diagnosis of valve (mal-)function and will be addressed in future studies.

Utilizing radiographic contrast agents, µPCCT analysis can also be employed to detect organic occlusions by determining void spaces in the contrast agent distribution. Provided this information, comparison to time resolved radiographic contrast agent flow data can consecutively be utilized to either develop analytical models or to train U-Net-based models, capable of identifying organic depositions, and other failure mechanisms, from radiographic contrast flow series.

As seen in Fig. 6 U-Net-based obstruction detection shows a clear dependency on contrast agent concentration and obstruction size. Note that the contrast agent concentrations investigated here correspond to clinically available Gadolinium contrast agents. Whereas for very low photon statistics (<100 open beam counts) the detection performance deteriorates, at higher statistics the performance saturates quickly. Also note that for the results shown in Fig. 6 the U-Net was trained using 1250 frames of mixed parameter sets (5 × 5 × 5, five different values for each of the above parameters), thus emulating strongly varying experimental conditions and corresponding non-optimal results. The resulting detection efficiency therefore must be considered non-optimal.

Fig. 7 shows the results of U-net obstruction detection (using 2500 simulated projections for training, and 2500 others for evaluation, both with randomly placed occlusions with nvolumes between 0.03 mm^3^ and 0.9 mm^3^). The median dice similarity coefficient (DSC) remains almost unaffected by the obstruction volume down to 0.15 mm^3^. For even smaller volumes an increasing number of outliers becomes apparent.

As shown in Fig. 8, at a detection threshold of 0.5 we obtain a localization specificity of >90% and sensitivity of ∼70%, at average DSC >80% and a false positive rate of <10%. With reference the flow measurements shown in Fig. 2, the expected dose load of the proposed procedure is <5 µSv.

## Conclusion

The high failure rate of CSF shunt valves warrants research of diagnostic methods which ideally are sufficiently time- and dose-efficient for regular prophylactic screening. The methods demonstrated above provide means to analyze CSF-shunt morphology and occurring failure mechanisms. The projection geometry used in our radiographic series is in principle clinically viable.

Currently there are no clinical imaging systems based on photon-counting technology which fit the desired geometry for in-vivo shunt analysis. However, available energy-integrating clinical systems, such as angiography systems or dental cone-beam CT scanners, are geometrically well suited for the task. Positioning the patient off-center in a dental cone-beam CT scanner, the projection magnification can be adapted and the CSF-SV can be centered in radiography. Furthermore, such system designs allow to image the patient’s head with inclination (as illustrated in Fig. 1), also allowing to analyze patency of the gravitational unit. Therefore, preceding the potential construction of a dedicated CSF-SV scanner with an optimized geometry and photon-counting technology, the utilization of such widely available systems appears promising for first clinical studies.

Our results demonstrate that radiographic series can determine the flow of a small bolus of a clinical contrast agent solution through a CSF-SV with high temporal and spatial resolution. First tests using U-Net-based analysis on single radiographies suggest that valve obstructions can be detected and localized precisely under the assumed conditions. Other indicators of valve patency, such as correct spring compression and proper mobility of the gravitational unit, can complement this diagnosis. The anticipated low dose load of the proposed method could enable its utilization in regular screening processes, allowing to detect and possibly also to counteract possible CSF-SV failures early on.

The proposed approach also has potential application fields in medical research and product development. For example, approaches to resolve obstructions of the valve, e.g. by lysis of coagulations, could be monitored in real time. Consequently, potential treatment options for failing shunt valves could be quickly investigated and optimized. Vice versa, by longitudinally observing the clogging behavior of CSF-SVs under laboratory conditions, optimization of valve designs to prevent obstructions can be undertaken efficiently. Here, the proposed methodology could complement computational fluid dynamics simulations, and also be utilized to calibrate such routines.

### Limitations and possible extensions of this work

A shortcoming of the present study is the lack of reference data from explanted malfunctioning CSF-SVs. Consequently, the assessment of U-NET-based valve diagnostics had to be based on synthetic data. However, the current pilot study demonstrates the ample diagnostic capabilities of the proposed approach. Verification of the presented methods based on explanted CSF-SVs needs to be performed in future studies.

Also, in our simulation study we did not consider superimposed patient morphology. This limitation however we consider non-critical since 1. landmark-based registration of the valve will hardly be affected by soft tissue background and 2. the U-NET flow analysis performs on digital subtraction radiography, which largely eliminates influence of static superimposed morphology.

It should be noted that the U-NET study in the current work was based on single projections for simplicity. Utilization of time series, as depicted in Fig. 2, will add complexity to the problem, but may also further increase the diagnostic capabilities of the approach.

Finally, it must be mentioned that the proposed approach requires the injection of a contrast agent into the shunt system, posing a possible threat of infection.

## Origin of the work

Division of Medical Physics, Department of Diagnostic and Interventional Radiology, University Medical Center Freiburg, Faculty of Medicine, University of Freiburg, Freiburg, Germany**,** Killianstrasse 5a, 79106 Freiburg**,** Germany.

## Funding information

This work was supported by the Research Commission of the Medical Faculty, University Freiburg, Germany through the grant “Neue präklinische Bildgebungsmodalitäten: Konventionelles und spektrales µCT”.

## Data sharing statement

All data generated and analyzed during the study are available from the corresponding author on reasonable request.

## Declaration of competing interest

This work was supported by a hardware loan of DECTRIS AG of Baden-Daettwil, Switzerland, who provided the photon-counting detectors employed in the current study. Martin Pichotka: Shareholder of speCTive GmbH, Freiburg, Germany. Moritz Weigt: Shareholder of speCTive GmbH, Freiburg, Germany. Christopher L. Schlett: Speaker Bureau by Siemens Healthineers and Bayer Healthcare. All other authors declare no competing interests.

## Appendix A Supplementary data

Supplementary video 1 Contrast flow measurement using Gd contrast agent. Radiographic frames were taken at 20Hz using an EIGER2X CdTe photon-counting detector. The color coding is analogous to Fig. 2.
